# Diabetic striatopathy: an updated overview of current knowledge and future perspectives

**DOI:** 10.1007/s40618-023-02166-5

**Published:** 2023-08-14

**Authors:** A. Arecco, S. Ottaviani, M. Boschetti, P. Renzetti, L. Marinelli

**Affiliations:** 1 Endocrinology Unit, Department of Internal Medicine and Medical Specialties, School of Medical and Pharmaceutical Sciences, University of Genova, 16132 Genoa, Italy; 2 Section of Geriatrics, Department of Internal Medicine and Medical Specialties, University of Genova, 16132 Genoa, Italy; 3 IRCCS Ospedale Policlinico San Martino, 16132 Genoa, Italy; 4 Department of Neuroscience, Rehabilitation, Ophthalmology, Genetics, Maternal and Child Health, University of Genova, 16132 Genoa, Italy

**Keywords:** Hyperglycemia, Hemichorea/hemiballism, Basal ganglia, CT hyperdensity, T1-weighted MRI hyperintensity

## Abstract

**Purpose:**

Diabetic striatopathy (DS) is a rare complication of poorly controlled diabetes mellitus (DM), characterized by hyperglycemia associated with chorea/ballism and characteristic reversible basal ganglia abnormalities on computed tomography (CT) and/or magnetic resonance imaging (MRI). We propose a narrative review of the literature on this topic, currently unknown to most, and about which physicians should be aware. We intend to summarize, critically review, and take to mean the evidence on this disorder, describing its typical features.

**Methods:**

We searched Pubmed for English-language sources using the following keywords in the title and the abstract: diabetic striatopathy, hyperglycemic non-ketotic hemichorea/hemiballism, chorea/hemichorea associated with non-ketotic hyperglycemia, diabetic hemiballism/hemichorea, chorea, hyperglycemia, and basal ganglia syndrome. We collected scientific articles, including case reports, reviews, systematic reviews, and meta-analyses from the years 1975 to 2023. We eliminated duplicate, non-English language or non-related articles.

**Results:**

Older Asian women are more frequently affected. Suddenly or insidiously hemichorea/hemiballism, mainly in the limbs, and high blood glucose with elevated HbA1c in the absence of ketone bodies have been observed. Furthermore, CT striatal hyperdensity and T1-weighted MRI hyperintensity have been observed. DS is often a treatable disease following proper hydration and insulin administration. Histopathological findings are variable, and no comprehensive hypothesis explains the atypical cases reported.

**Conclusion:**

DS is a rare neurological manifestation of DM. If adequately treated, although treatment guidelines are lacking, the prognosis is good and life-threatening complications may occur occasionally. During chorea/hemiballism, we recommend blood glucose and HbA1c evaluation. Further studies are needed to understand the pathogenesis.

## Introduction

Diabetic striatopathy (DS) is a rare complication of poorly controlled diabetes mellitus (DM), mainly type 2, characterized by hyperglycemia associated with chorea/ballism and/or reversible characteristic basal ganglia abnormalities on CT and/or MRI brain imaging. Nomenclature in scientific literature is very heterogeneous: “diabetic striatopathy” [1–3], “hyperglycemic non-ketotic hemichorea/hemiballism” [4–6], “chorea/hemichorea associated with non-ketotic hyperglycemia” [7–10], “diabetic hemiballism/hemichorea" [11, 12], “chorea, hyperglycemia, basal ganglia syndrome” [13–15]. “Diabetic striatopathy” definition was introduced about ten years ago [16] and is probably the most complete and up-to-date. It describes a relatively uncommon condition of hyperglycemia associated with chorea/ballism and basal ganglia hyperdensity on computed tomography (CT) and/or hyperintensity on T1-weighted nuclear magnetic resonance imaging (MRI). According to a recent systematic review [2], DS would also include patients with a hyperglycemic condition associated with even one of the following: (1) chorea/ballism; (2) striatal hyperdensity on CT or hyperintensity on T1-weighted MRI. Furthermore, a possible classification of DS has been recently proposed [17].

This narrative review aims to provide an updated and inclusive overview of relevant literature about DS, focusing mainly on mostly obscure and controversial pathogenetic mechanisms.

## Literature search methods

We conducted a literature search on the Pubmed database using the following keywords in the title and the abstract: “diabetic striatopathy”, “hyperglycemic non-ketotic hemichorea/hemiballism”, “chorea/hemichorea associated with non-ketotic hyperglycemia”, “diabetic hemiballism/hemichorea”, “chorea, hyperglycemia, and basal ganglia syndrome”. We collected scientific articles, including case reports, reviews, systematic reviews, and meta-analyses published in English language from the years 1975 to 2023. We eliminated duplicate, non-English language or non-related articles.

## Diabetic striatopathy: rare but undervalued disease

Bedwell [18] described this rare syndrome several decades ago. Several case reports have been published, mainly describing older women of Asian origin with uncontrolled type 2 DM, defined as high glycated haemoglobin (HbA1c) levels, who typically present with acute-onset hemichorea.

The prevalence of DS is about 1 in 100,000, which is probably extensively underestimated because most physicians are not aware of this condition that could be misdiagnosed as intracerebral haemorrhage due to hyperdensity on CT [19]. Among patients with poorly controlled type 2 DM (HbA1c > 10%) hospitalised for any cause, about 0.58% had DS, while the percentage rises to 1.2% among those hospitalised for neurological symptoms [20]. A retrospective study provided a first prevalence value of DS in Italy, which was lower (0.16%) than in Shafran's studies. In addition, knowledge of HbA1c value and age may help predict which patients are at increased risk of developing DS [21].

Up to 90% of the reported cases occurred in the Asian population [7], while according to a more recent review [2] such percentage is lower (71.6%). Data initially indicated that the syndrome was characteristic of this ethnic population, suggesting this predominance is related to a genetic predisposition or inadequate diabetes control system in underdeveloped countries [7]. Several reports of DS in Caucasian [15, 20, 22, 23] and Hispanic [13, 24, 25] populations ruled out both of the above hypotheses. The higher prevalence of DS in women has also been questioned: a male-to-female ratio of 1:1.76 [7] and 1:1.7 [2] were reported. Female predominance is probably due to underdiagnosis in males rather than a real difference [20]. Changes in Gamma Amino Butyric Acid (GABA) or dopamine receptors in post-menopausal women following estrogenic changes have also been considered [22]. Hemichorea does not only affect elderly type 2 diabetics with non-ketotic hyperglycemia with an average onset age of 67.6 years old [2] but several cases are reported during type 1 DM in the pediatric population [26–31].

Although DS is a rare complication of DM, it is now considered the second most common cause of hemichorea/hemiballism, right after cerebrovascular events involving basal ganglia (both ischemic and hemorrhagic stroke) [32, 33], and the most common in the metabolic cause group [6].

## Basal ganglia damage as a cause of movement disorders

The basal ganglia (BG) are a group of interconnected subcortical nuclei in the ventromedial part of the cerebral hemispheres, primarily involved in motor control (such as posture, tone, and movement) and motor learning, as well as in cognition and affective control [34]. The term “basal ganglia” in a narrower sense refers to deep nuclei in the cerebral hemispheres [caudate nucleus (CN), nucleus accumbens (Acb), putamen (Put) and globus pallidus (GP)], while the “related nuclei” are made up of structures located in the diencephalon [subthalamic nucleus of Luys (SNT)], mesencephalon [substantia nigra of Sommering (SN), ventral tegmental area] and the pons (pedunculopontine nucleus) [35]. The term “striatum or corpus striatum” comprises CN, Put, Acb and GP.

According to their function, the BG can be classified as input nuclei, output nuclei and intrinsic nuclei. The input nuclei include CN, Put and Acb: they receive incoming information from the cortex, thalamus and nigral nuclei. The output nuclei include the internal segment of the globus pallidus (GPi), and the substantia nigra pars reticulata (SNr): they send basal ganglia information to the thalamus. The intrinsic nuclei, such as the external segment of the globus pallidus (GPe), the STN, the substantia nigra pars compacta (SNc) and the ventral tegmental area, are located between the input and output nuclei [35]. Cortical and thalamic efferent information enters the striatum, is processed and via the output nuclei projects mainly to the thalamus, which in turn projects to the cerebral cortex (mainly to the frontal lobe), forming a cortico-basal ganglia-thalamo-cortical loop [34].

DeLong first proposed the model of basal ganglia functioning [36]: it involves two parallel circuits in which the cerebral cortex and the SNc project to the input nuclei, giving rise to an indirect inhibitory and a direct excitatory pathway. This model is still valid, but according to Calabresi et al. it should be revised to integrate more recent scientific findings [37]. A simplified description of basal ganglia functioning will be provided here to allow the readers to understand the alterations that occur during diabetic striatopathy [35].

The input nuclei (CN, Put and Acb) contain two different types of projection neurons, also called medium-sized spiny neurons (MSNs), because of their structural characteristics. All striatal MSNs are inhibitory neurons and use GABA as a neurotransmitter. These neurons can be further subdivided, based on their projection targets, into two groups: MSN neurons innervating the GPe, expressing the dopamine receptor subtype 2 (D2R) and giving rise to the indirect pathway (striato-GPe-STN-GPi/SNr), and MSN neurons projecting to the output nuclei (GPi and SNr), expressing the dopamine receptor subtype 1 (D1R) and giving rise to the direct striatopallidal pathway. All ipsilateral and contralateral cortical areas project to the input nuclei via glutamatergic projections (cortico-striatal pathway). The input nuclei also receive dopaminergic afferents from the SNc (nigrostriatal pathway). Nigrostriatal neurons project to both types of striatal MSN neurons: dopaminergic input exerts an excitatory effect on D1R MSNs (direct pathway neurons) and an inhibitory effect on D2R MSNs (indirect pathway neurons). The output nuclei also consist of tonically active inhibitory GABAergic neurons. They receive inhibitory GABAergic inputs from the MSN D1R neurons (direct pathway), and excitatory glutamatergic inputs from the STN (indirect pathway). In turn, the output nuclei innervate some thalamic neurons, mainly ventral anterior and lateral thalamus nuclei, which project glutamatergic axons to the motor cortex. The GPe, like the GPi, consists of GABAergic neurons that project to the output nuclei. It represents the second synaptic station of the indirect pathway and receives GABAergic afferents from MSN D2R. The STN consists mainly of glutamatergic projection neurons with excitatory effects: it receives GABAergic afferents from the GPe and projects to the output nuclei. Finally, the SN consists of two portions: the pars reticulata, part of the output nuclei, and the pars compacta, containing dopaminergic neurons that project to the input nuclei.

Therefore, to summarize, the classical model of basal ganglia function comprises an indirect pathway that inhibits and a direct pathway that facilitates movement production. The indirect inhibitory pathway originates in the striatal MSN D2R neurons, which inhibit GPe, leading to activation (following disinhibition) of STN, which activates the ventral anterior and lateral thalamic nuclei and then the motor cortex. The direct excitatory pathway originates in the striatal MSN D1R neurons, which inhibit output nuclei, leading to disinhibition of the anterior and lateral ventral thalamic nuclei and consequent activation of the motor cortex.

Basal ganglia damage can cause either akinetic-rigid movement disorders (such as Parkinson's disease) or hyperkinetic movement disorders [34]. Chorea and ballism belong to hyperkinetic movements that are neither rhythmic nor stereotyped. Bizet provided definitions of chorea and ballism and their lateralised counterparts, hemichorea and hemiballism [13]. Chorea is characterized by irregular involuntary movements, random in pattern, not rhythmic or repetitive, small in amplitude and mainly distal. Hemicorea, on the other hand, involves one body side and is caused by lesions in the contralateral striatum [8]. Ballism is defined as a set of involuntary, constantly variable, non-rhythmic and non-suppressible movements of large amplitude in the proximal portion of the limbs. Hemiballism, in turn, is characterized by involuntary unilateral limb movements resulting from a lesion in the contralateral subthalamic nucleus and adjacent structures [8]. These movements usually appear during wakefulness, are exacerbated by stressful conditions and disappear during sleep [29].

## Histopathological findings are few and inconclusive

Few histological findings are reported in the literature, both because of the type of tissue as errors in sampling nervous tissue can have irreversible sequelae and because of the site of biopsy sampling—i.e. basal ganglia—which is difficult to reach. On the other hand, few subjects underwent autopsy, and basal ganglia sampling was not always included. All reports showed the same heterogeneity observed in both clinical and neuroimaging studies. All documented the presence of astrocytosis [16, 38–41]. Four of these studies also described the presence of a macrophage infiltrate, haemorrhage with extravasation of erythrocytes, focal microhemorrhages, hemosiderin deposits and hemosiderin-containing macrophages [16, 39–41], with a delay in one case of 33 days after the neuroimaging findings [40]. Gliosis, hyalinosis of blood vessels and many gemistocytes were found in a biopsy performed 60 days later [38]. Other pathological findings reported were: infarction areas [40], multiple small foci of tissue necrosis [16, 39], lumen narrowing of the arterial wall with fibrosis [39, 41] and calcifications in the infarct area [40].

There is still no consensus about which histological findings are characteristic of DS, which can explain neuroimaging changes and what the aetiology might be. However, because of the transient nature of this syndrome, careful analysis of the timing of onset and evolution of imaging changes is necessary, and there is no doubt that histological studies can make an outstanding contribution to the knowledge of what causes clinical and neuroimaging changes.

## What are the causes of the involuntary movements?

As to why hyperglycemia causes hemichorea/hemiballism, two main theories have been put forward, which are not mutually exclusive but probably related: the metabolic and vascular theories.

**Fig. 1:**
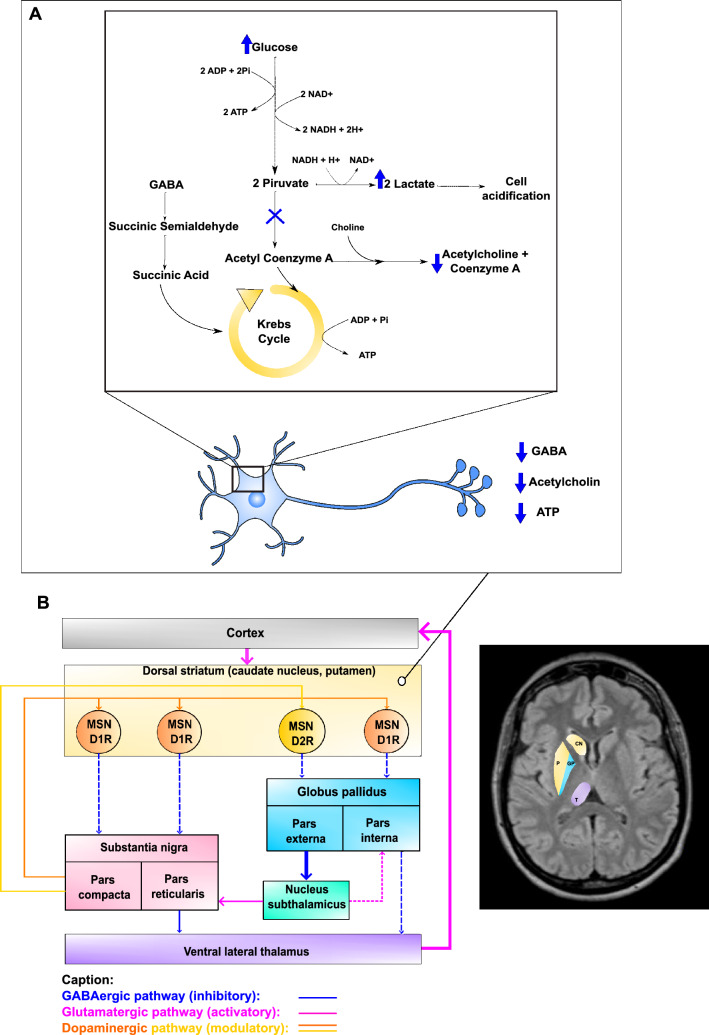
Metabolic theory. **A** Biochemical changes within basal ganglia neurons during non-ketotic hyperglycemia. **B** Neuroanatomical diagram of the connections between cerebral cortex and basal ganglia and visual representation of caudate nucleus (CN), putamen (P), globus pallidus (GP) and thalamus (T) on an axial T1-weighted-FLAIR MR image. Thicker arrows indicate connection becoming hyperactive because of striatal dysfunction, while dotted arrows indicate connection becoming hypoactive. Interruption of GABAergic transmission from the striatum to the external globus pallidus (GPe) [medium spiny neurons expressing dopamine D2 receptor (MSN D2R)] leads to an abnormal increase in the activity of the GPe neurons, which in turn exerts an inhibitory action on the subthalamic nucleus (STN). Increased inhibition of the STN, secondary to the increase in GPe activity, leads to a loss of control over internal globus pallidus (GPi). On the other hand, the GABAergic transmission is interrupted [medium spiny neurons expressing dopamine D1 receptor (MSN D1R)] from the striatum to the GPi, which receives this afferent and the excitatory inputs from the STN. The imbalance between the indirect excitatory and direct inhibitory pathways, resulting in a loss of inhibitory control by the GPi/substantia nigra (SNr) on the thalamus, leads to a disinhibition of the motor thalamus

According to the metabolic theory [7], non-ketotic hyperglycemia could be one of the possible mechanisms involved (Fig. 1). During hyperglycemia, cerebral metabolism shifts to the anaerobic pathway of glucose metabolism, with consequent inactivation of the Krebs cycle. As an alternative energy source, the brain metabolises GABA into succinic acid. Nevertheless, the energy from GABA only provides 10–40% of what the basal ganglia require, leading to metabolic acidosis. GABA and acetate are rapidly depleted, and so acetylcholine synthesis is reduced. Thus, the depletion of both GABA and acetylcholine in the basal ganglia concomitant with metabolic acidosis and the lack of energy production can lead to basal ganglia dysfunction and clinical signs. On the one hand, there is a disinhibition of the subthalamic nucleus and thus an excessive activation of the motor cortex by thalamic projections [42, 43]. On the other, during ketotic hyperglycaemia, GABA can be re-synthesized from ketone bodies, explaining why chorea is less frequent in these patients, even though some cases have been reported [44].

However, this hypothesis makes it challenging to explain various aspects observed. Firstly, in most patients, the clinical manifestations have a unilateral onset, although hyperglycemia is a systemic condition. Secondly, choreic movements persist even after blood sugar levels are normalised [45]. Thirdly, some patients develop chorea after rapidly correcting hyperglycaemia [13, 22, 46]. The onset of delayed chorea could be attributed to a temporary compensation of GABA depletion due to high levels of ketone bodies [46], but this needs further evidence. Some do not present ketoacidosis [13, 22], while among patients with ketotic hyperglycemia, none showed delayed hemichorea after correcting hyperglycaemia [47]. Fourthly, chorea can also occur during hypoglycaemia [48–50] and ketotic hyperglycaemia [44]. Several possible pathophysiological mechanisms of brain damage associated with hypoglycaemia have been hypothesised, some of which are shared with hyperglycaemia. One of these mechanisms could be anoxia due to cytotoxic oedema resulting from abrupt decreases in energy and ion pump activity induced by glucose deprivation [51]. Another would be decreased basal ganglia blood flow (and reduced glucose delivery) and increased perfusion of the thalamus contralateral to the body side affected by chorea [7, 52, 53]. Lastly, the deficiency of GABA and acetylcholine would also begin in the hyperglycemic period and become more evident during hypoglycemia secondary to insulin treatment [7]. Repeated episodes of hypoglycemia have been associated with cognitive impairment and other neurological complications [54]. Thus, non-ketotic hyperglycemia could be only one of the possible mechanisms of chorea.

According to vascular theory, ischemia is the pathogenetic mechanism underlying the clinical manifestations [55]. Kim [56] maintains that the leading cause of the hemicoretic/hemiballic movements is hypoperfusion in the striatum, which leads to a dysfunction of the medium-sized spiny GABAergic projection neurons. On the one hand, the interruption of GABAergic transmission from the striatum to the external globus pallidus (GPe) [medium spiny neurons expressing dopamine D2 receptor (MSN D2R)] leads to an abnormal increase in the activity of the GPe neurons, which in turn exerts an inhibitory action on the subthalamic nucleus (STN). Increased inhibition of the STN, secondary to the increase in GPe activity, leads to a loss of control over internal globus pallidus (GPi). On the other hand, the GABAergic transmission is interrupted [medium spiny neurons expressing dopamine D1 receptor (MSN D1R)] from the striatum to the GPi, which receives this afferent and the excitatory inputs from the STN. The imbalance between the indirect excitatory and direct inhibitory pathways, resulting in a loss of inhibitory control by the GPi/substantia nigra (SNr) on the thalamus, leads to a disinhibition of the motor thalamus. Brain Single Photon Emission Computed Tomography (SPECT) shows decreased blood perfusion of the basal ganglia and increased perfusion of the thalamus contralateral to the site of clinical manifestations, although not statistically significant when compared with healthy controls [56].

It is not clear what causes hypoperfusion. Epidemiologically, the most susceptible population is elderly patients with poorly controlled DM, who are thought to suffer from diabetic vasculopathy [57]. Therefore, these are predisposed patients who are affected by a second factor leading to hypoperfusion: it may be the increase in blood viscosity, secondary to dehydration, induced by non-ketotic hyperglycemia, which manifests with acanthocytosis, thus leading to ischemia [58]. Alternatively, the same metabolic and osmotic process that deforms red blood cells also could affect neuronal membranes, thus compromising neuronal function. So, acanthocytosis could only represent an epiphenomenon of the pathogenetic mechanism. Cerebral hypoperfusion results from increased cerebrovascular resistance due to the higher water content in the brain during hyperglycaemia or from a loss of flow regulation caused by altered metabolism [55].

Although the vascular theory is convincing in several respects, it leaves a few questions open. Firstly, it cannot explain the development of bilateral chorea in the case of unilateral imaging alterations. Secondly, most patients with DS present with transient clinical manifestations with a benign course, suggesting partial striatal damage but not a complete infarction as the cause.

A final pathogenetic mechanism put forward by some authors is bleeding, especially microhemorrhages, due to hyperintensity on T1-weighted MRI [7] and hyperdensity on CT. However, only a few histological findings reported the presence of focal microhemorrhages [16, 39–41]. Further studies are needed to consider microbleeding as a possible pathogenetic mechanism.

## What causes neuroimaging changes?

Several hypotheses have been formulated to explain the imaging abnormalities: calcifications [40], petechial haemorrhage [41], deposition of paramagnetic substances [59], destruction of myelin [60] and infarction with reactive astrocytosis [38, 40].

Although the calcification theory cannot be completely ruled out based on imaging abnormalities alone, such rapid resolution of lesions makes haemorrhagic genesis much more likely. The acute clinical onset also argues in favour of haemorrhage rather than a chronic process such as calcium salt deposition. Finally, tissue density values of hyperdensities are generally between 40 and 50 Hounsfield units (HU). This value is like that of petechial haemorrhage, whereas calcifications are close to 80 HU [61].

The petechial haemorrhage hypothesis was the first put forward [62] and has enjoyed considerable success. The neuroradiological pattern of DS, consisting of hyperdensity on CT scan, hyperintensity on T1-weighted MRI and hypointensity on T2, may suggest the presence of haemorrhage and intracellular methemoglobin. However, parenchymal hematomas or a massive hemorrhagic transformation in the context of infarction may be excluded because of the absence of mass effect or oedema and the absence of an initial MRI signal compatible with the presence of deoxyhemoglobin. Therefore, petechial haemorrhage without oedema is the only bleeding that reflects these features. In addition, the accelerated oxidation of haemoglobin to meta oxidation of haemoglobin to methemoglobin by free radicals can occur in ischemic areas undergoing rapid reperfusion, thus explaining the absence of the typical acute hemorrhagic MRI appearance. The presence of extravascular deposits of hemosiderin in the putamen suggests that hyperglycemia can induce a transitory dysfunction of the blood–brain barrier, which is responsible for erythrocyte extravasation [41]. Therefore, striatopathy would be one of the multiple aspects assumed by diabetic vasculopathy.

A few years later, Taguchi [63] took up this theory, arguing that the finding of simultaneous hypointensity in T2* images could further support the idea of petechial haemorrhage with hemosiderin deposits, but other studies have not confirmed this. Indeed, some aspects do not agree with the typical haemorrhagic presentation: the HU of the lesion (lower in petechial than massive haemorrhage) and, more importantly, the persistence for several months or even years after onset [38].

Based on the latter observation, Shan [38] agrees that the high density on CT scan is due to petechial haemorrhage rather than calcium deposition, a phenomenon not supported by many bioptic findings [40] and, above all, it is unlikely to reverse. However, they exclude that this phenomenon can also explain the T1 hyperintensity because of the long persistence of the hypersignal and the existing mismatch with the areas of hyperdensity on CT. Therefore, Nath [40] attributes the phenomenon to the proliferation of a particular cell species: gemistocytes, reactive, swollen astrocytes that appear during an acute brain injury and chronic diseases such as subacute sclerosing panencephalitis or epilepsy. Gemistocytes are localised along the course of axons and can persist for years, undergoing progressive shrinkage. Shortening T1 relaxation time may result from the abundant protein matrix within the cytoplasm of swollen gemistocytes, producing electrostatic interactions that restrict the movement of water molecules, as in one reported case of gemistocytic astrocytoma [64]. In support of this theory, gemistocytic astrocytes have been described in autopsy reports of patients with hemichorea/hemiballism [16, 38–41].

The idea that gemistocytes can alter the relaxation time of molecules through the induction of manganese-containing enzymes following transient ischaemia was subsequently put forward [59, 65]. Striatocapsular ischaemia caused by temporary occlusion of the internal carotid artery or middle cerebral artery by cardiogenic emboli could produce T1 hyperintensity in the basal ganglia. Prolonged ischaemia causes T1 hypointensity and T2 hyperintensity in an entire hemisphere, corresponding histologically to pan-necrosis of brain tissue, with early cavitation and macrophage infiltrate. In contrast, transient ischaemia causes T1 hyperintensity and T2 hypointensity seven days later; this new neuroradiological finding has been called “Delayed Ischemic Hyperintensity” on T1-weighted MRI. Biopsy demonstrated incomplete ischaemia with selective neuronal loss and gliosis in the corresponding lesion but with relative preservation of structural integrity of the brain tissue (i.e., microvacuolation was absent). However, it was unlikely that the selective neuronal loss alone with gross preservation of brain tissue characteristic of incomplete infarction would affect X-ray transmission and the magnetic field. Therefore, biochemical factors, such as the deposition of paramagnetic compounds (characterised at the atomic level by magnetic dipoles that align with the magnetic field), i.e. metal ions, molecular oxygen or free radicals, have been suggested to underlie the altered relaxation times. The shortening of T1 relaxation time results, at least in part, from the induction in the mitochondria of reactive astrocytes of a free radical scavenger, manganese-superoxide dismutase (Mn-SOD) and glutamine synthetase (GS), also containing manganese [66]. Spectroscopy and immunohistochemical studies have shown increased manganese concentrations in the striatum concomitant with changes in MRI signal and immunoreactivity to Mn-SOD and GS in astrocytes. With its subsequent rapid improvement, the acute onset of DS would support a vascular aetiology, such as transient infarction. However, the presence of manganese-filled gemistocytes does not explain the CT changes, leading some researchers to speculate that the findings on CT, MRI imaging, and associated hemichorea-hemiballism may reflect different pathologic mechanisms.

Finally, another possible mechanism involves hematic hyperviscosity. This is supported by restricted diffusion, evidenced by diffusion-weighted imaging (DWI) hyperintensity and reduced apparent diffusion coefficient (ADC) values in hyperintense areas at T1-weighted MRI [67]. This finding suggests the presence of cytotoxic oedema and transient due to blood hyperviscosity. It has been observed that with proper treatment, DWI abnormalities such as cytotoxic oedema can regress, similar to what has been observed in stroke for ischaemic penumbra. Recently, significantly reduced ADC values have been confirmed in the basal ganglia, appearing T1 hyperintense [68].

Hyperviscosity is closely linked to plasma hyperosmolarity. This can cause a reduction in cerebral flow (confirmed by SPECT), leading to transient ischaemia but not infarction [69]. Supporting elements are laboratory findings of plasma hyperosmolarity during choreoathetosis, variations on T2-weighted MRI that could reflect variations in osmolarity and high levels of myo-inositol. This osmolyte regulates astrocyte volume: glucose 6-phosphate excess results in increased inositol synthesis in hyperglycaemia.

The prolonged hypoperfusion of the basal ganglia, found for more than four months, could explain the long latency in the disappearance of T1 hyperintensity but not the sudden improvement in symptoms [70]. Another possible explanation could be a demyelinating mechanism [60], but histological studies have no evidence.

As a result, there is currently no certainty as to what causes the alterations in CT and MRI and whether this is common to both. It would be helpful to follow patients with DS by serial imaging over time to appreciate the changes and obtain autopsy specimens and conduct specific basal ganglia sections in patients with DS and diabetic subjects without neurological manifestations to compare which histopathological findings are common and which are specific to DS.

A recent case report showed that DS and intracerebral haemorrhage (ICH) might simultaneously appear similar at brain CT [6]. Although DS could be more easily misdiagnosed as ICH due to the hyperdense on CT imaging, differential diagnosis is facilitated by the absence of hypodensity around the hyperdense area, a sign of perihaematoedema [71], and the lack of increase in the CT attenuation value (up to values of 80–100 HU), a mark of the presence of haemoglobin within a haematoma [72].

## Typical neuroimaging findings include increased striatal signal at CT and MRI-T1 and reduced at MRI-T2 scans

**Fig. 2:**
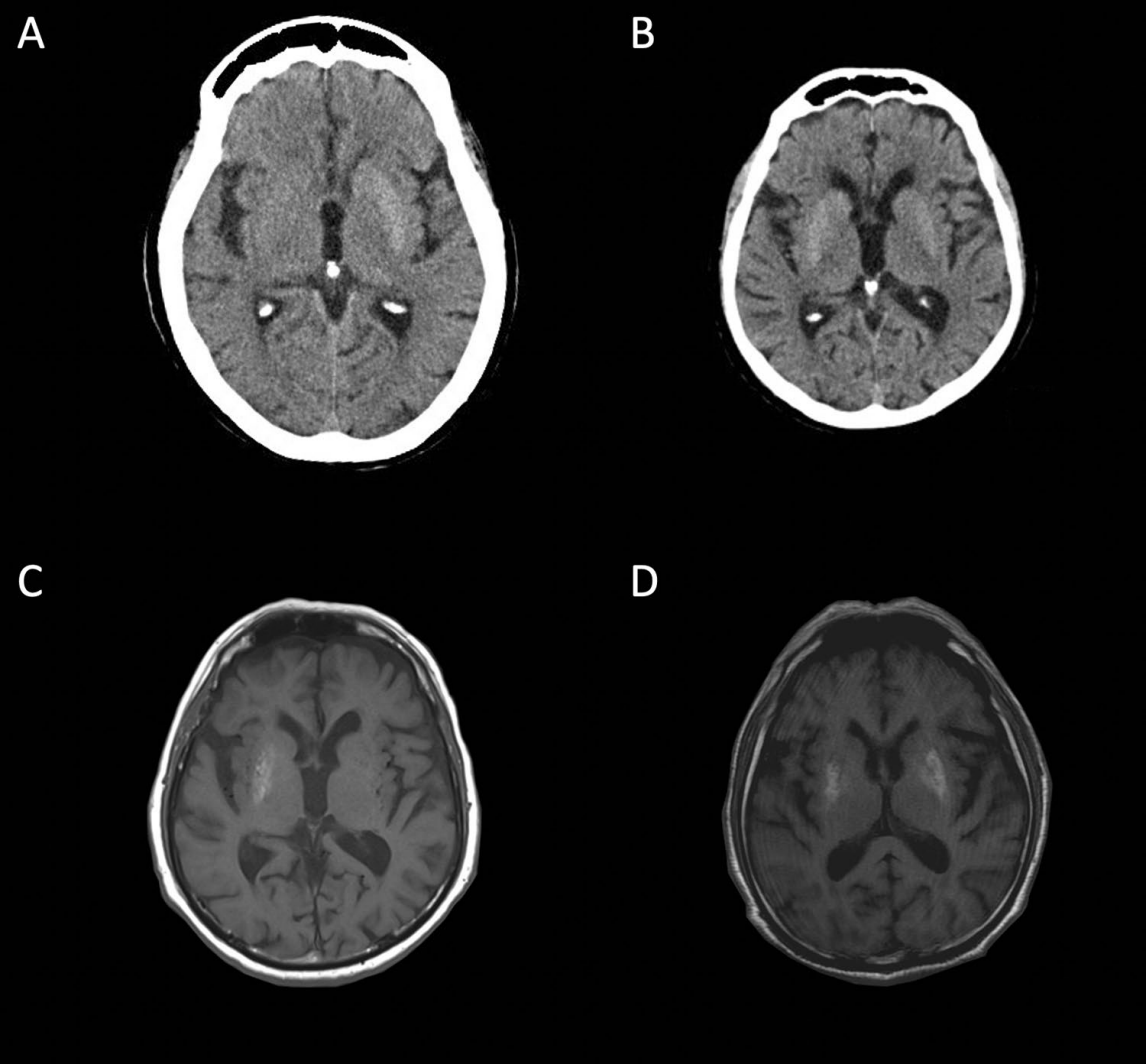
Typical neuroimaging of diabetic striatopathy. **A**, **B** Axial CT scan: contralateral caudate nucleus and putamen (**A**) and bilateral striatal hyperdensity (**B**). **C**, **D** Axial T1-weighted MR image: contralateral (**C**) and bilateral pallidal-putaminal (**D**) hyperintensity can be observed

Typical neuroradiological changes are striatal hyperdensity on CT scan and hyperintensity on T1-weighted MRI (Fig. 2). Isolated contralateral putamen is the most frequent striatal abnormality, followed by simultaneous involvement of contralateral caudate nucleus and putamen with constant sparing of the internal capsule [2, 7, 68]. Involvement of all three striatal components (caudate nucleus, putamen and globus pallidus) is possible [2]. However, the association between neuroimaging changes and symptoms varies widely. Although the limbs are more frequently clinically involved (in order of frequency arm-leg, arm-leg-face and isolated arm), two cases of isolated facial hemichorea with oral dyskinesia and grimacing are also reported [73, 74].

Furthermore, about one-sixth of “mismatch” (17.5%) and “incompatibility” (14.6%) between CT and MRI findings, defining mismatch as the complete absence of basal ganglia abnormalities in one but not in the other, or incompatible locations of striatal anomalies between the two imaging techniques [2]. MRI proved more sensitive, as MRI abnormalities were found in patients with negative CT. In contrast, no cases with positive CT were found in the presence of negative MRI.

There are exceptions to the typical DS alterations. Firstly, although the clinical features are compatible with choreic movements, neuroimaging may not show any characteristic DS lesions [3, 75]. Secondly, although the clinical manifestations are unilateral, the imaging lesions may appear bilaterally [22] as in patients with bilateral chorea [9, 24, 76, 77].

In most cases, neuroradiological changes are detected after the appearance of hemichorea since CT or MRI are usually performed after symptoms appear. While latency between stroke and hemichorea appearance on average ranges between 1 and 7 days in vascular hemichorea, accurate information concerning DS is lacking [78]. Cases where brain imaging (CT) has been incidentally performed before symptoms onset are few, reporting a variable latency ranging from 40 to 14 days [38, 79, 80]. Also, the timing of the resolution of CT/MRI alterations is very variable: MRI alterations could still be evident six years later [38].

Using gradient echo-weighted (T2*-GRE) and susceptibility-weighted imaging (SWI) MRI sequences may offer further clues to narrow the field of differential diagnostics. In DS, such scans have revealed striatal hypointensity [16, 81]. This finding could indicate a petechial haemorrhage with hemosiderin deposits [63] or an accumulation of paramagnetic materials (iron [82] or manganese [59]). However, the finding of hypointensity has also proved controversial and inconsistent, as other studies have found no signal abnormalities in T2 [38] and T2* [69]. Therefore, striatal hypointensity on T2-weighted imaging should not be a criterion for DS diagnosis.

Similarly, DWI sequence findings have also been variable: some reported restricted diffusion in the putamen [83, 84] while others did not [16, 40]. A possible explanation could be the onset of acute putaminal dysfunction, secondary to a hyperglycemic or hyperosmolar insult and associated with some degree of Wallerian degeneration of the putaminal internal white matter.

Functional imaging techniques, such as technetium-99 m Hexamethylpropyleneamine Oxime SPECT and Fluorodeoxyglucose-Positron Emission Tomography (FDG-PET), have also been used, demonstrating basal ganglia hypoperfusion (preceded by temporary hyperperfusion due to autoregulation deficit) [56, 85, 86] and glucose hypometabolism, supporting the theory of metabolic failure in the injured area [7, 67, 87]. A lengthy follow-up made it possible to ascertain the persistence of striatal hypoperfusion even more than three years after the onset of the involuntary movements [85].

Similarly, MRI spectroscopy revealed increased lactic acid, acetate and lipids levels, while the N-acetylaspartate/creatine ratio decreased and the choline/creatine ratio increased [16, 69, 70, 88]. These findings indicate the presence of ATP depletion caused by the onset of anaerobic glycolysis, and neuronal dysfunction, both of which are compatible with incomplete infarction. Finally, increased choline content suggests active cell proliferation in the context of the putamen, e.g. by gemistocytes. In two studies, angioMRI disclosed middle cerebral artery stenosis, possibly causing basal ganglia hypoperfusion [89] or oozing-type findings, supporting microhemorrhage's role in the pathogenesis of DS [90].

## Irregular firing of internal globus pallidus is related to choreic movements

Exciting data emerged from recording the neuronal discharge pattern of the neurons of the internal globus pallidus belonging to a patient suffering from DS and subjected to a pallidotomy operation [91]. The tracing revealed a lower discharge frequency and, above all, irregular compared to that of parkinsonian patients in the 'off' phase (without ongoing dyskinesias); similar findings were found by Hashimoto in the GPi of a patient affected by lacunar infarcts of the striatum [92]. This suggested that hyperactivity of the direct pathway may be responsible for GPi inhibition, thus causing disinhibition of thalamocortical projections and, ultimately, choreic movements. However, the paradoxical success of some neurosurgical procedures, such as pallidotomy and deep brain stimulation of the GPi, which completely inhibit the firing of these neurons, may suggest that the real culprit of choreic movement appearance is the irregular discharge pattern, with frequent pauses, rather than the low-frequency firing (Fig. 1B).

## Choreic and ballic movements appear acutely and are frequently lateralised

Often chorea and ballism are associated with each other: as stated above, both belong to hyperkinetic movements that are neither rhythmic nor stereotyped. Most patients with DS (96.6%) have type 2 DM, among which about the one-sixth present with newly diagnosed diabetes [2], thus suggesting that DS could be one of the first possible presentations of DM. The most typical presentation is access to the emergency department for acute/subacute onset of chorea/ballism, typically in an older female subject with a long history of poorly controlled diabetes. In more than 90% of cases, the onset is unilateral limb chorea/ballism [2, 7], whereas only 9.7% present with bilateral involvement [9, 24].

Before the onset of hemichorea/hemiballism, various not-neurological prodromal symptoms have been observed, such as chest [83] and shoulder pain [93]. Among prodromal symptoms, hemiparesis [94], gait disturbances [94], dizziness [25, 46], vertigo [73], lethargy [95], confusion [58], and coma [96] have been described, suggesting that chorea is sometimes a delayed clinical manifestation of a contralateral striatal lesion [16].

The onset and distribution vary from patient to patient. In some cases, these movements may start abruptly and suddenly, in others insidiously, moving from a small to a large amplitude. At the same time, they may occur intermittently or continuously. Cases of chorea with a diffuse pattern have been reported, most commonly from the upper to the lower limbs [73], more rarely vice versa [11].

Cases of DS without hemichorea have also been described: clinical manifestations vary from disturbances of consciousness to focal neurological signs, such as limb weakness, dysarthria and dysphagia [97].

## High glycated haemoglobin and absent ketones are frequent laboratory findings

During DS, blood glucose levels and HbA1c are variable, generally both high, although euglycemic [13, 98] or even hypoglycemic values [99] are sometimes found. The mean blood glucose level was 481.5 mg/dL (169–1264 mg/dL), and HbA1c levels were 14.4% (9.9–19.2%). Of the 71 patients tested for ketone bodies, 81.7% were negative for both ketonemia and ketonuria, indicating that DS is manifested as non-ketotoacidemic hyperglycemia in most cases [7]. Recent studies in patients with DM suggest that low glycemic control can contribute to basal ganglia alterations and the development of DS [100], while normal HbA1c levels may prevent or delay it [101].

Moreover, some cases of delayed onset of DS have been described [13, 22, 98, 102], with the onset of clinical manifestations ranging from one week [22] to one month after the hyperglycemic episode, even after reasonable glycemic control [98]. The mechanism of hemichorea/hemiballism with euglycemia at presentation may be the delayed ischemic effect of hyperglycaemia [98]. Thus, it is necessary to remember DS as a cause in patients with hemichorea/hemiballism even weeks after optimal control of hyperglycemia since this is a correctable condition.

## Involuntary movements may last many months and require pharmacological or even surgical treatment

Reducing blood glucose levels, achieved by adequate hydration and administration of insulin, is the cornerstone of DS treatment [1]. Resolution of involuntary movements is highly variable, from a few days to about ten months after correction of hyperglycaemia, with an average of approximately six months for complete improvement; some patients show only partial improvement between 3 months and 5.6 years after diagnosis [7]. However, only a quarter of patients achieve resolution of clinical symptoms with glycaemic control alone, as most patients require additional anti-choreic drugs [2]. These belong mainly to five classes: antipsychotics, dopamine-depleting agents, benzodiazepines, anticonvulsants, and serotonin reuptake inhibitors. Among typical antipsychotics, haloperidol [7, 38] was the most widely used, followed by chlorpromazine, sulpiride, pimozide, and tiapride [7]. Atypical antipsychotics, such as risperidone [13] and quetiapine [103], have also been used. Since antipsychotic drugs can cause tardive dyskinesias, their use must be carefully considered. Tetrabenazine [83] and reserpine [38] are dopamine-depleting agents and act by blocking the presynaptic monoamine transporter. Benzodiazepines, such as diazepam [7, 86] and clonazepam [7, 38], enhance GABA receptor inhibitory activity. Anticonvulsants, such as sodium valproate [7] and topiramate [103], and selective serotonin reuptake inhibitors, like escitalopram [1], have also been used. Combined regimes are sometimes reported [1, 50]. The time required for symptoms to disappear is, on average, significantly shorter (2 days) in patients on glycaemic control alone than in those on additional anti-choreic medication (14 days), reflecting a less severe disease in the former [2].

For patients with symptoms refractory to medical therapy, invasive approaches have been attempted with moderate success: pallidotomy [104], ventrolateral thalamotomy [7], transcranial magnetic stimulation [105], internal globus pallidus deep brain stimulation [106]. Some patients relapsed after stopping anti-chorea drugs over two months and two years after the first episode of chorea [7]. In most patients, the chorea recurred on the same side previously affected, but some patients, who initially had a unilateral onset, developed bilateral chorea. A relatively high recurrence rate of about 20% was observed even after the resolution of the neuroimaging abnormalities [2], thus pointing to the need for regular follow-up independent of neuroimaging results (Table 1).

**Table 1:** Features of diabetic striatopathy

Definition	Hyperglycaemic condition associated with even one of the following: (1) chorea/ballism; (2) striatal hyperdensity on CT or hyperintensity on T1-weighted MRI
Epidemiology	
Prevalence	1:100 000 (underestimated)^a^
Ethnicity	Asian more frequently, but also Caucasian and Hispanic
Male female ratio	1:1.7^b^
Histopathology	Variable: astrocytosis, macrophage infiltrate, haemorrhage with extravasation of erythrocytes, focal microhaemorrhages, haemosiderin deposits and haemosiderin-containing macrophages, gliosis, hyalinosis of blood vessels, gemistocytes, infarction areas, multiple small foci of tissue necrosis, lumen narrowing of arterial wall with fibrosis, calcifications in the infarct area
Imaging	
CT	Striatal hyperdensity
MRI	T1-weighted: striatal hyperintensity T2-weighted: variable (> hypointensity) SWI-weighted: variable (> hypointensity) DWI-weighted: variable (> restricted diffusion)
CT-MRI concordance	Possible mismatch (17.5%)^b^ and incompatibility (14.6%)^b^
99mTc HMPAO SPECT	Basal ganglia hypoperfusion
FDG-PET	Glucose hypometabolism
Clinic	
Type of movement	Haemichorea-heamiballism
Type of movement (in order of frequency)	Arm-leg, arm-leg-face, isolated arm, isolated face
Onset of movement	Variable: suddenly or insidiously
Laboratory test	
Blood glucose level	Hyperglycaemia (mean 414 mg/d)^b^
Glycated haemoglobin	Elevated (mean 13.1%)^b^
Ketone bodies	Negative (81.7%)^b^
Treatment	
Correction of hyperglycaemia	Hydration and insulin
Control of movement (if correction of hyperglycaemia is not sufficient)	Typical antipsychotics: haloperidol, chlorpromazine, sulpiride, pimozide, tiapride Atypical antipsychotics: risperidone, quetiapine Dopamine-depleting agents: tetrabenazine, reserpine Benzodiazepines: diazepam, clonazepam Anticonvulsants: sodium valproate, topiramate Serotonin reuptake inhibitors: escitalopram
Prognosis	Good and curable

*99mTc HMPAO* technetium Tc 99m hexamethylpropyleneamine oxime, *DWI* diffusion weighted imaging, *FDG* fluorodeoxyglucose, *SWI* susceptibility weighted imaging

^a^Ondo 2011[19]

^b^Chua et al. 2020[2]

## Conclusions

DS is a rare neurological manifestation in patients with DM. It is curable, and if adequately treated, the prognosis is good, although treatment guidelines are lacking, and life-threatening complications may occur occasionally. During chorea/hemiballism, we recommend blood glucose and HbA1c evaluation. In addition, a detailed history, objective examination, and neuroimaging are essential. Typical clinical manifestations are haemichorea/haemiballism, unilateral hyperkinetic movements that are neither rhythmic nor stereotyped, the former small in amplitude and mainly distal, the latter large in amplitude and mostly proximal. Typical findings are hyperdensity on CT and hyperintensity on T1-weighted MRI in contralateral basal ganglia, mainly putamen. However, there is only sometimes a clinical-neuroradiological and CT-MRI correspondence. Based on current knowledge, the former is difficult to explain, so further retrospective studies are needed. Conversely, a higher sensitivity of MRI could explain CT-MRI incompatibility. Early treatment, through glycaemic control and administration of anti-chorea drugs, can reduce the impact on patient's quality of life and minimize the risk of other neurological complications. Further studies are needed to understand the pathogenesis. Histological studies, primarily through autopsy samples, are essential: biopsy samples are challenging and dangerous, as sampling errors can have permanent consequences. Similarly, retrospective studies are crucial to increase the knowledge about clinical-neuroradiological and CT-MRI concordance, epidemiology, and therapy.
